# Architecture and evolution of the lithosphere beneath the northern South China sea: Implications for plate-edge lithospheric thinning and rifting

**DOI:** 10.1016/j.fmre.2025.03.002

**Published:** 2025-03-11

**Authors:** Qiang Ma, Sheng-Ping Qian, Yi-Gang Xu, Fan Yang

**Affiliations:** a Southern Marine Science and Engineering Guangdong Laboratory (Guangzhou), Guangzhou 511458, China; b State Key Laboratory of Deep Earth Process and Resources (DEEPER), Guangzhou Institute of Geochemistry, Chinese Academy of Sciences, Guangzhou 510640, China

**Keywords:** South china sea, Lithosphere structure, Continental lithospheric thinning and rifting, Water, Mantle transition zone

## Abstract

Passive margins formed by continental rifting and oceanic spreading exhibit a wide range of characteristics, many of which remain poorly understood. In this study, we investigate the continent-ocean transition (COT) of the South China Sea (SCS) to elucidate the tectonic-magmatic processes involved in continental thinning and rifting. We present new in situ analyses of water, chemical, and Sr isotopic compositions of volcanic glasses and plagioclases, alongside the ages and Hf isotope compositions of zircons from the SCS. Our isotopic data reveal significant inter- and intra-crystalline disequilibria in plagioclase phenocrysts, suggesting a complex history of mantle-derived magmas interacting with the continental crust. Geochemical variability in the plagioclases and the presence of old inherited zircons indicate that mantle magmas were contaminated by crustal material. The ages (ranging from Cretaceous to Archean) and Hf isotopic signatures of the zircons are similar to those found in the Cathaysian block of Southeast China, supporting the presence of continental lithosphere beneath the northern SCS. The COT is characterized by a sharp juxtaposition of continental crust and igneous oceanic crust, and its structural features differ from the classic magma-poor or magma-rich passive-margin models. The X_H2O_ conditions inferred for basalt generation during continental rifting and SCS spreading imply that the underlying mantle beneath the SCS was hydrous, which may have triggered the weakening and removal of the lithospheric root, probably due to the multiple subductions of oceanic plates since the Cretaceous. Our findings provide key evidence for the role of hydro-induced lithospheric weakening associated with nearby subduction zones in the processes of continental thinning and breakup in the SCS.

## Graphical abstract

**Figure:**
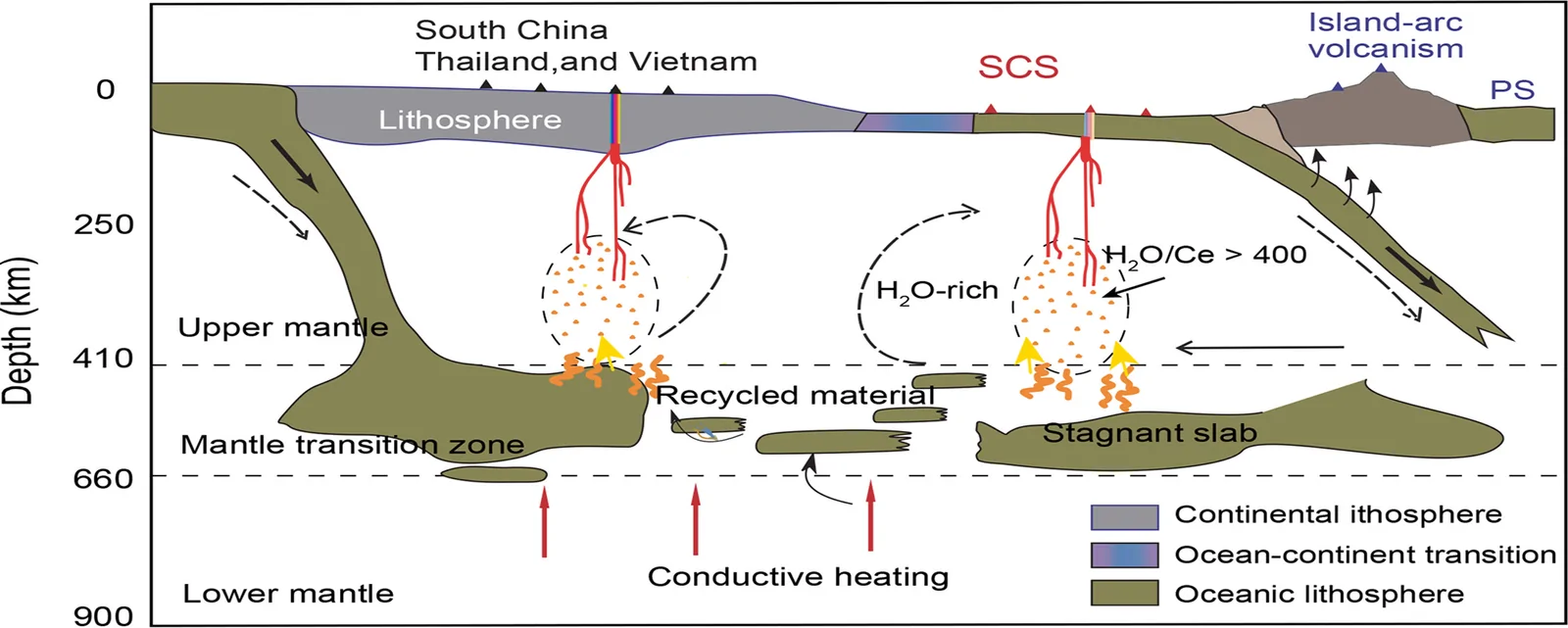

## 1 Introduction

Passive margins developed as a result of continental rifting and ocean-floor spreading. Based on the volume of magmatism during the syn-rift and syn-breakup, rifted continental margins are usually classified as volcanic rifted (magma-rich) and non-volcanic (or magma-poor) margins [1,2]. The continent-ocean transition (COT), which marks the boundary between the thinned continental crust and the onset of the oceanic crust, is a critical feature of rifted continental margins [3]. This zone holds essential insights into the mechanisms of continental breakup and the evolution of different rift margin types [4,5]. However, the precise nature and structure of the COT remain difficult to delineate, often due to ambiguous seismic interpretations and a paucity of drilling and geochemical data [6,7]. The South China Sea (SCS), one of the largest marginal seas in the western Pacific (Fig. 1), has long been considered a small-scale analogue to the Atlantic, characterized by the development of broad passive margins to the north and south [8]. The multiphase rifting and opening of the SCS have resulted in varying structural styles along the passive margin [[9], [10], [11]]. Despite extensive seismic studies and drilling investigations, the precise nature and evolution of the SCS passive margin remain contentious. The northern SCS passive margin has been classified as magma-poor, possibly hosting exhumed serpentinized mantle in its distal regions [12]. Seismic data indicate high-velocity anomalies near the COT in the northeastern SCS, potentially linked to upwelling and serpentinization of the mantle [13]. However, results from the International Ocean Discovery Program (IODP) Expeditions 367/368, which drilled across the COT in the northern SCS, challenge this view [14]. These findings support the existence of a narrow COT in this region, characterized by a rapid transition from continent to ocean without mantle exhumation [10]. As a result, the nature of the SCS passive margin has been debated, with models ranging from magma-poor [12], intermediate [5,9,10], and even magma-robust [11].

**Fig. 1:**
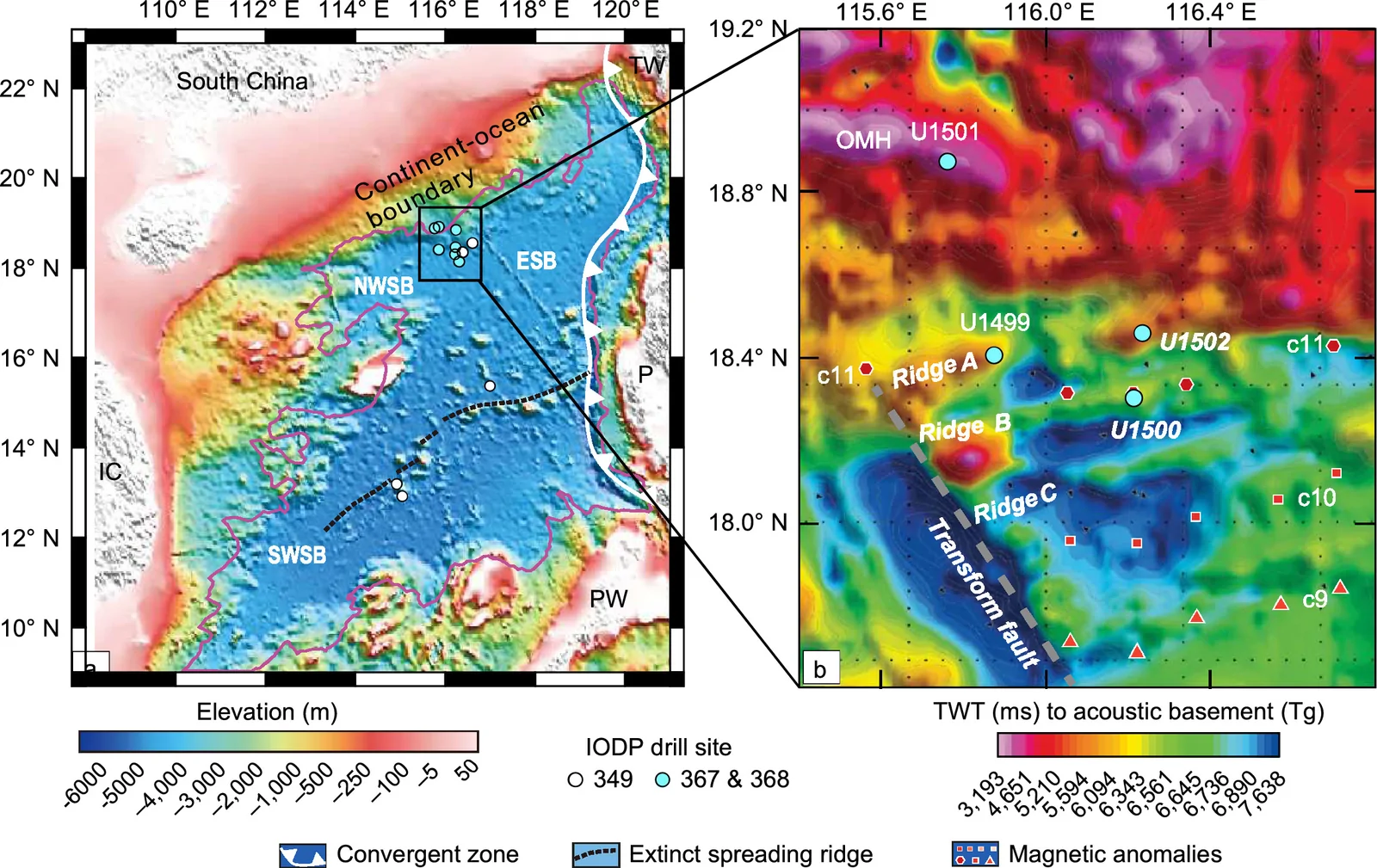
(a) Main tectonic features of the SCS showing the location of IODP Sites [10,16] and oceanic magnetic anomalies [21]. Ocean Sub-basins: NWSB: Northwest Sub-basins, ESB: East Sub-basins, SWSB: Southwest Sub-basins. Continental domains: IC: Indochina, PW: Palawan, P: Philippines, TW: Taiwan. (b) Zoomed map of the top acoustic basement (Tg reflector) [10] on the northern SCS margin segment showing the distinct topographic features of IODP drilling sites. The two sites on Ridge A (U1499 and U1502) are separated by 40 km distance.

IODP Expedition 367/368 focused on a transect through the COT in the northern SCS margin, drilling into the oceanic basement (Fig. 2) [10,15,16]. The recovered igneous basement from the distal margin of the northern SCS, obtained from Holes U1500 and U1502, provides crucial material for investigating the nature of this unique passive margin. Here, we present detailed analyses of volatile, chemical, and isotopic compositions of volcanic glasses and feldspars, along with the ages and Hf isotope signatures of zircon extracted from basaltic rocks recovered from Sites U1500 and U1502 on the northern SCS margin (Fig. 2). These data offer new insights into the petrological characteristics of the COT, which, when integrated with prior geophysical and drilling data, enhance our understanding of the processes that shaped the continental lithosphere before the formation of the SCS.

**Fig. 2:**
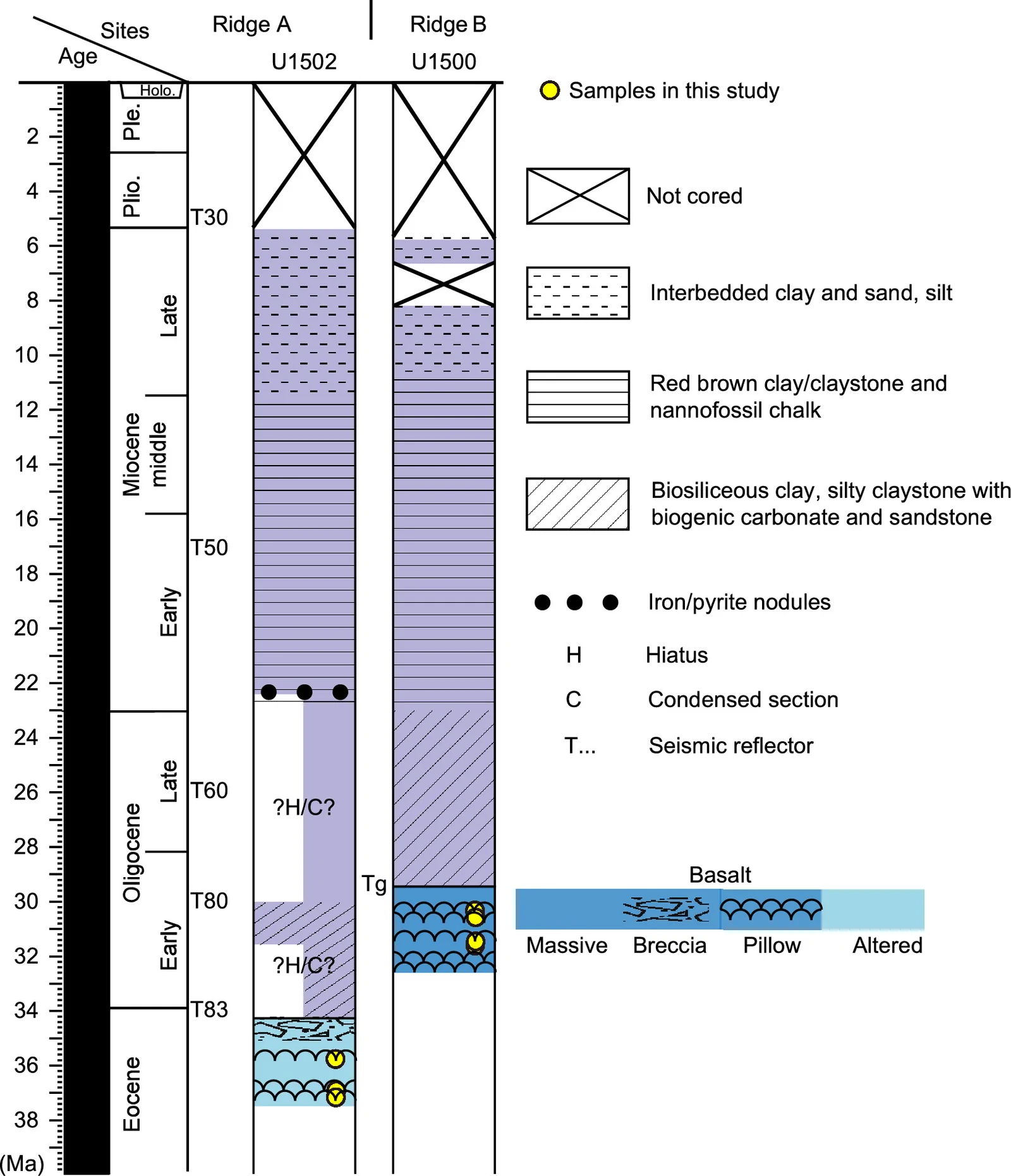
**Summary chart of IODP drilling results of Sites 1500 and 1502**. Ages based on core samples analyzed for calcareous nannofossils, foraminifers, diatoms, and ostracods; lithostratigraphy from detailed core descriptions following the GTS1243 geological timescale [10].

## 2 Geological setting and samples

The SCS is situated at the junction of the Eurasian, Indo–Australian, and Philippine–Pacific plates (Fig. 1) [[17], [18], [19], [20]]. The SCS oceanic basin, comprising several sub-basins (e.g., the northwest sub-basin (NWSB), east sub-basin (ESB), and southwest sub-basin (SWSB); Fig. 1), formed as a result of continental rifting, seafloor spreading and ridge jump between the early Oligocene and middle Miocene (33–15 Ma) [21,22]. The rifting process responsible for the formation of the SCS migrated from north to south and from east to west [14,23]. Despite a well-established temporal framework, the precise mechanisms behind the basin's opening and evolution remain unclear. Recent petrological, geochemical, and geophysical studies, alongside geodynamic modeling, have shown that the evolution of the SCS and its associated magmatism are closely linked to the complex subduction systems surrounding the basin [19,24,25].

The COT zone along the northern SCS margin (17°6′–19°2′ N, 115°2′ E–117°0′ E) is characterized by distinct basement highs, with an outer margin high (OMH) at the landward edge, followed seaward by three parallel basement ridges (A, B, and C) (Fig. 1b) [10,16]. This zone transitions from the continental OMH to the steady-state oceanic crust of Ridge C. The basalt samples analyzed in this study were recovered from the COT zone at IODP Sites U1500 and U1502 on the northern SCS margin.

At IODP Site U1502, located along Ridge A (Fig. 1), a total of 181.79 m of crystalline basement (basaltic lavas with pillow structures) was recovered between 739.16 and 920.95 m below the seafloor (mbsf) [10]. The basalt sequence at Site U1502 represents the oldest in-situ oceanic crust drilled to date, with ages predating the early Oligocene (∼34 Ma), as determined by SCS crustal seismology, magnetic anomalies, and biostratigraphy [10,15]. Site U1500, located approximately 80 km seaward of the OMH along Ridge B, recovered 150 m of basement basalts from 1379 to 1529 mbsf (Figs. 1, 2). Nannofossils of Oligocene age from sediment layers intercalated with the lavas are consistent with the alignment of magnetic chron C11n (∼29.5 Ma) with Ridge B (Figs. 1, 2) [10,15].

## 3 Analytical methods

### 3.1 In situ U-Pb dating and HF isotope analyses of zircon

Due to the limited occurrence of zircons in basalts, there is ongoing concern about potential natural or anthropogenic contamination of the samples. To prevent contamination, strict precautions were taken during the zircon collection. Prior to processing, all worksites and equipment were thoroughly cleaned to prevent any potential contamination. To avoid contamination during crushing and pulverizing, a high voltage pulse power fragmentation device was applied to the sample. Density separation with heavy liquid (methylene iodide) and magnetic separation with a Frantz magnetic separator were then employed, followed by hand-picking under a binocular microscope to ensure purity. The zircons exhibit varied morphologies, ranging from euhedral to subhedral, with some larger (∼200 µm in length) and elongated crystals (Fig. 3). Cathodoluminescence (CL) images were taken for all zircons in the State Key Laboratory of Marine Geology at Tongji University.

**Fig. 3:**
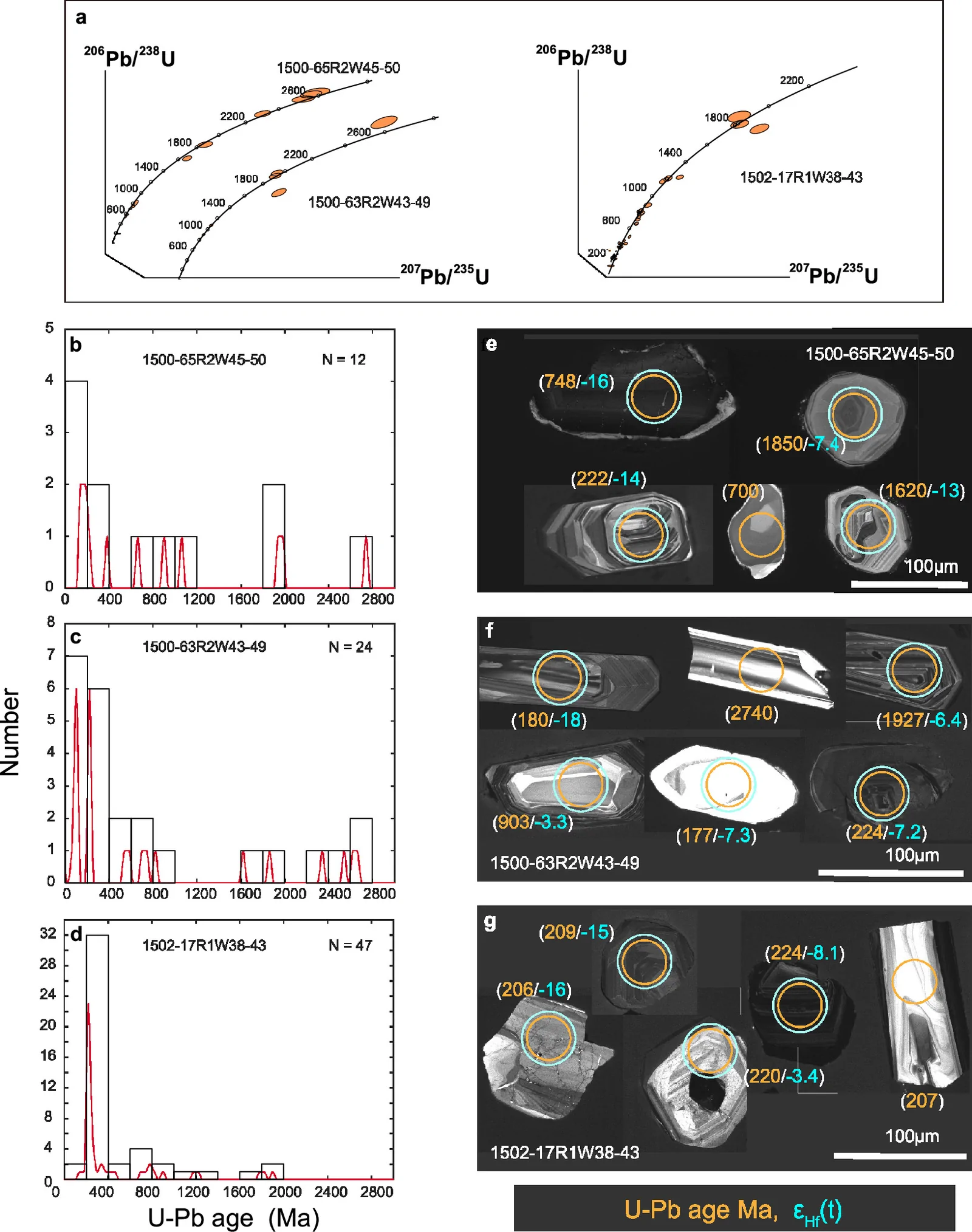
**Concordia plots and CL/BSE images of zircons in South China Sea lavas**. ^206^Pb/^238^U ages are taken for zircons in this study. Zircon crystals vary in morphology and CL texture from euhedral to subhedral.

Zircon U-Pb dating and trace element analysis were performed using LA-ICP-MS (a RESOlution M-50 laser ablation system and a Thermo Element 2 instrument) at the Guangzhou Institute of Geochemistry, Chinese Academy of Sciences (GIGCAS), China. Each zircon crystal surface was cleaned with ethanol to remove potential contamination before analysis. The analysis spot size was approximately 32 µm in diameter, with a repetition rate of 5 Hz. Each analysis included a 30 s background acquisition (gas blank), followed by 50 s of data acquisition from the sample. To monitor external uncertainties, the standard zircons Plešovice and GJ-1 were alternately analyzed as unknowns. Common Pb was corrected using the Excel program ComPbCorr#_151 [26]. Off-line calculations of isotopic ratios were conducted using GLITTER software. Each analysis included a 20–30 s background acquisition and 50 s data collection from the sample. The U-Pb dating and trace element results for the zircon standards Plešovice, Tanz and GJ-1 are presented in Table S1.

Hafnium isotope compositions in zircon crystals were determined using a Neptune Plus MC-ICP-MS (Thermo Scientific) coupled with a RESOlution M-50 193 nm laser ablation system at the GIGCAS. Zircons selected for Hf isotope analysis were those previously analyzed for U-Pb dating. The typical laser spot size for Hf analysis was 44 µm, with an ablation time of 26 s, a repetition rate of 10 Hz, and a laser energy density of 10 *J*/cm². Corrections for isobaric interference of ^176^Lu and ^176^Yb on ^176^Hf must be made precisely. Applying an exponential law and a ^176^Yb/^171^Yb of 0.897145, ^171^Yb was measured and corrected for the isobaric interference of ^176^Yb [27]. The interference of ^176^Lu on ^176^Hf was corrected by measuring ^175^Lu, and applying mass bias corrections based on the assumption that Lu and Yb behaved similarly (βLu = βYb), with a ^176^Lu/^175^Lu ratio of 0.02655 [28]. Initial ε_Hf_ values were calculated using a ^176^Lu decay constant of 1.865 × 10^–11^ year^−1^ [29], with a CHUR ^176^Hf/^177^Hf of 0.282785 and a ^176^Lu/^177^Hf of 0.0336 [30]. ^176^Hf/^177^Hf of standard 91,500, Plešovice, Jilin, and GJ-1 zircons are presented in Table S4.

### 3.2 Major and trace element analyses of plagioclases and glasses

Major element compositions of plagioclase and glass samples were determined using a JEOL JXA-8230 electron probe-analyzer at the State Key Laboratory of Marine Geology, Tongji University. The analyses were performed with an accelerating voltage of 15 kV, a beam current of 10 nA, and a beam diameter of approximately 3 µm. Elemental analyses were calibrated using mineral standards from SPI Company, with matrix corrections applied using the ZAF procedure, as implemented by JEOL.

In situ trace element analysis of glass samples was conducted using a 193 nm excimer laser ablation system (GeoLas 2005) coupled with an Agilent 7500 ICP-MS at the GIGCAS. Isotopes with minimal interference were selected for each element using factory-provided software. Analytical conditions were optimized by fine-tuning the carrier and make-up gas flows during the ablation of NIST SRM 610 to achieve maximum signal intensity for ^208^Pb, while maintaining low ThO/Th (0.1%–0.3%) and Ca^2+^/Ca^+^ ratios (0.4%–0.7%) to minimize interferences from oxides, argides, and doubly charged ions [31].

Each analysis consisted of a 30-second gas blank acquisition, followed by 50 s of data collection from the sample. Data processing—including background subtraction, signal integration, time-drift correction, and quantitative calibration—was carried out using the ICPMSDataCal software [31]. Analyses of reference glasses (BCR-2 G and BHVO-2 G) confirm that the analytical precision and accuracy are better than 10% (Table S3).

### 3.3 In situ SR isotope analyses of plagioclases and glasses

All in-situ Sr isotope analyses in this study were performed on a Neptune Plus MC-ICP-MS (Thermo Scientific), coupled with a RESOlution M-50 193 nm laser ablation system (Resonetics) at the GIGCAS. To enhance instrumental sensitivity, a small flow of N_2_ (2 ml L^-1^) and an X-skimmer cone were used in the interface. Isotope signals were detected using Faraday cups in static mode. All isotope signals are detected using Faraday cups in static mode. Laser ablation parameters were as follows: beam diameter, 112–155 µm; repetition rate, 9 Hz; energy density, approximately 4 *J* cm^-2^. Helium (800 ml min^-1^) was employed as the carrier gas. Each analysis consisted of 250 cycles with an integration time of 0.262 s per cycle. The first 30 s were used for gas blank measurement with the laser off, followed by 30 s of laser ablation for sample signal collection with the laser on. During analyses, the gas blank signals for ^83^Kr and ^88^Sr were consistently below 2.5 mV and 0.5 mV, respectively. Interferences from ^84^Kr and ^86^Kr on ^84^Sr and ^86^Sr were corrected by subtracting the gas blank from the raw time-resolved signal intensities. ^85^Rb was used to correct the interference of ^87^Rb on ^87^Sr with a natural ^85^Rb/^87^Rb = 2.593 [32]. The mass bias of ^87^Sr/^86^Sr was normalized to ^86^Sr/^88^Sr = 0.1194 with an exponential law. During the analysis, ^87^Sr/^86^Sr values of 0.70436 ± 0.00010 (2SD) and 0.70350 ± 0.00005 (2SD) were obtained for standard PZH (plagioclase) and NKT-1 G (glass), respectively, consistent within error with previously reported results (Table S2) [33,34].

### 3.4 Volatile analysis by sims

Volatile element compositions (H_2_O, Cl and S) of glasses were determined using the NanoSIMS Cameca 50 L at the Department of Terrestrial Magnetism, Carnegie Institution of Washington, following the protocol outlined in Hauri et al. [35]. Samples were analyzed using a rastered Cs⁺ primary beam with a current of 15–25 nA and an accelerating voltage of 10 kV. Prior to measurements, a 20-second presputtering was performed to clean the sample surface. After five minutes of pre-sputtering, the instrument cycled through the following masses: ^16^O^1^H, ^30^Si, ^31^P, ^32^S, and ^35^Cl. Data collection consisted of five cycles per analysis, with signals recorded by an electron multiplier, and dead-time correction was applied automatically. The basalt glass standard ALV-519–4–1 was measured every ten analyses to monitor analytical precision, yielding 0.176 wt.% H_2_O, 1020 ppm S, and 59.7 ppm Cl, in close agreement with published values (Table S5). Blank measurements of Suprasil and Herasil glass were also repeatedly performed to assess background levels, which were < 0.005 wt.% H_2_O, < 0.1 ppm S, and < 0.5 ppm Cl (Table S5).

## 4 Results

### 4.1 Zircon compositions

U-Pb dating and Lu-Hf isotope analysis were conducted on zircon grains separated from SCS Sites U1500 and 1502 basalts. Remarkably, the zircon population from SCS basalts is dominated by old inherited zircons, with ages spanning from the Cretaceous to the Archean (66–2740 Ma) (Figs. 3, 4). The age distribution reveals a prominent peak at 220 Ma, 750 Ma, and 1850 Ma (Fig. 4). These exotic or inherited zircons exhibit a broad range of εHf(t) values, from −18 to −3.3 (Fig. 4).

**Fig. 4:**
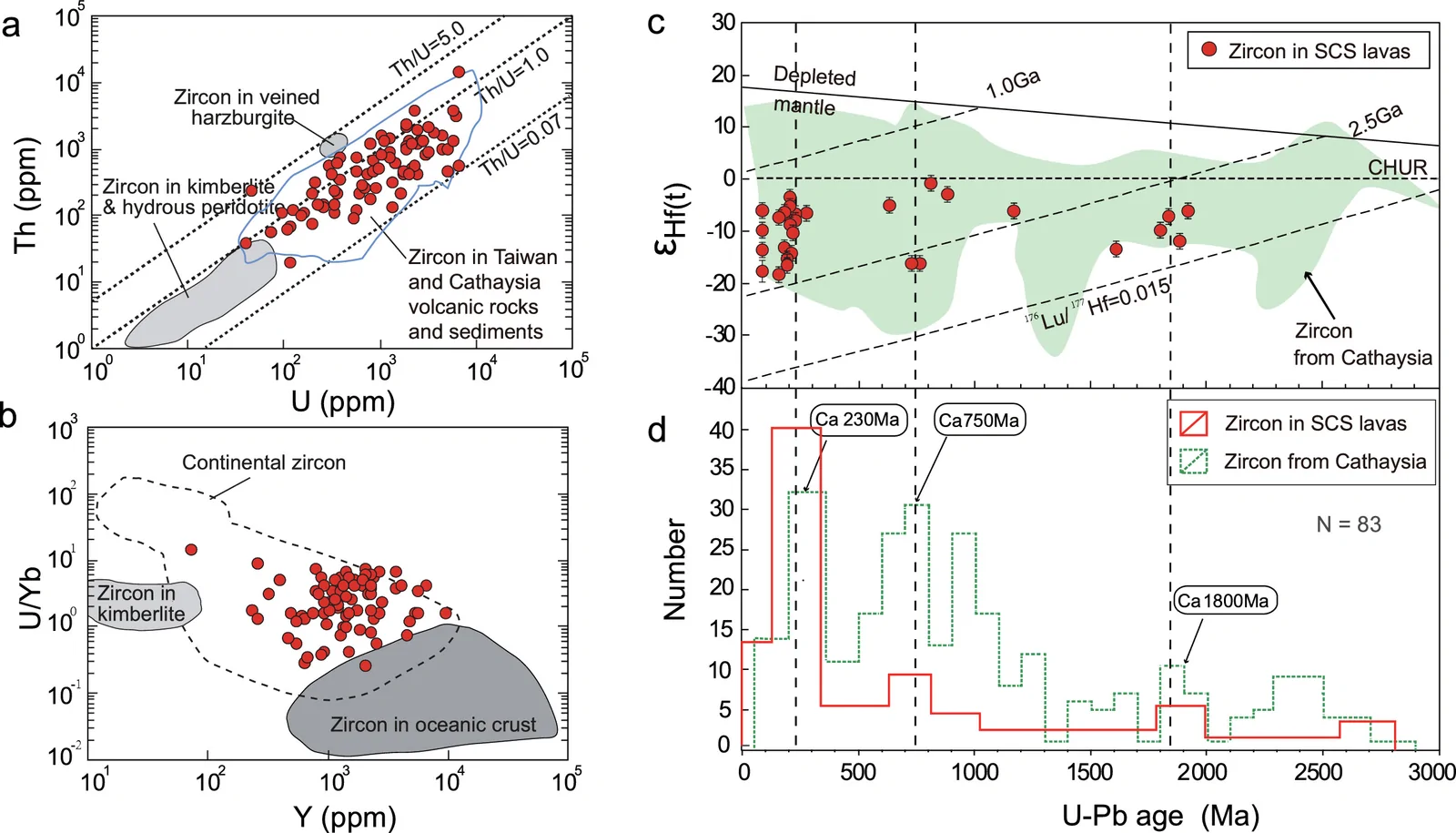
**Discriminant diagrams for zircon origin, and Hf isotopic compositions and age distribution of zircons in South Chin Sea lavas**. (a) Th versus U. (b) U/Yb versus Y, with zircons from South China Sea rocks showing crustal affinities. For comparison, the zircons from continental and oceanic crust, mantle peridotite and kimberlite [46], and Cathaysia Block volcanic rocks and sediments [45,54,70] are plotted. (c) Plot of ε_Hf_ (t) versus U-Pb age. (d) U-Pb age histogram. ^206^Pb/^238^U ages are used for zircons. The zircons in volcanic rocks and sediments from Cathaysia are plotted for comparison [45,54,70].

### 4.2 Plagioclase compositions

Sites U1500 and 1502 basalts are typically porphyritic and primarily contain plagioclase phenocrysts (Fig. 5). A large number of plagioclases from Site 1500 basalts display a core–rim normal zoning textural feature (Fig. 5a, b). Plagioclase phenocrysts are usually euhedral to sub-euhedral (Fig. 5). Most plagioclase phenocrysts with zoning textures have relatively narrow rims (Fig. 5a, b). The core zones of this type of plagioclase are overall more calcic (higher An 80.4–88.6, An = molar Ca/(Ca + Na + *K*)) than the rims (lower-An 67.7–75.8) (Fig. 5).

**Fig. 5:**
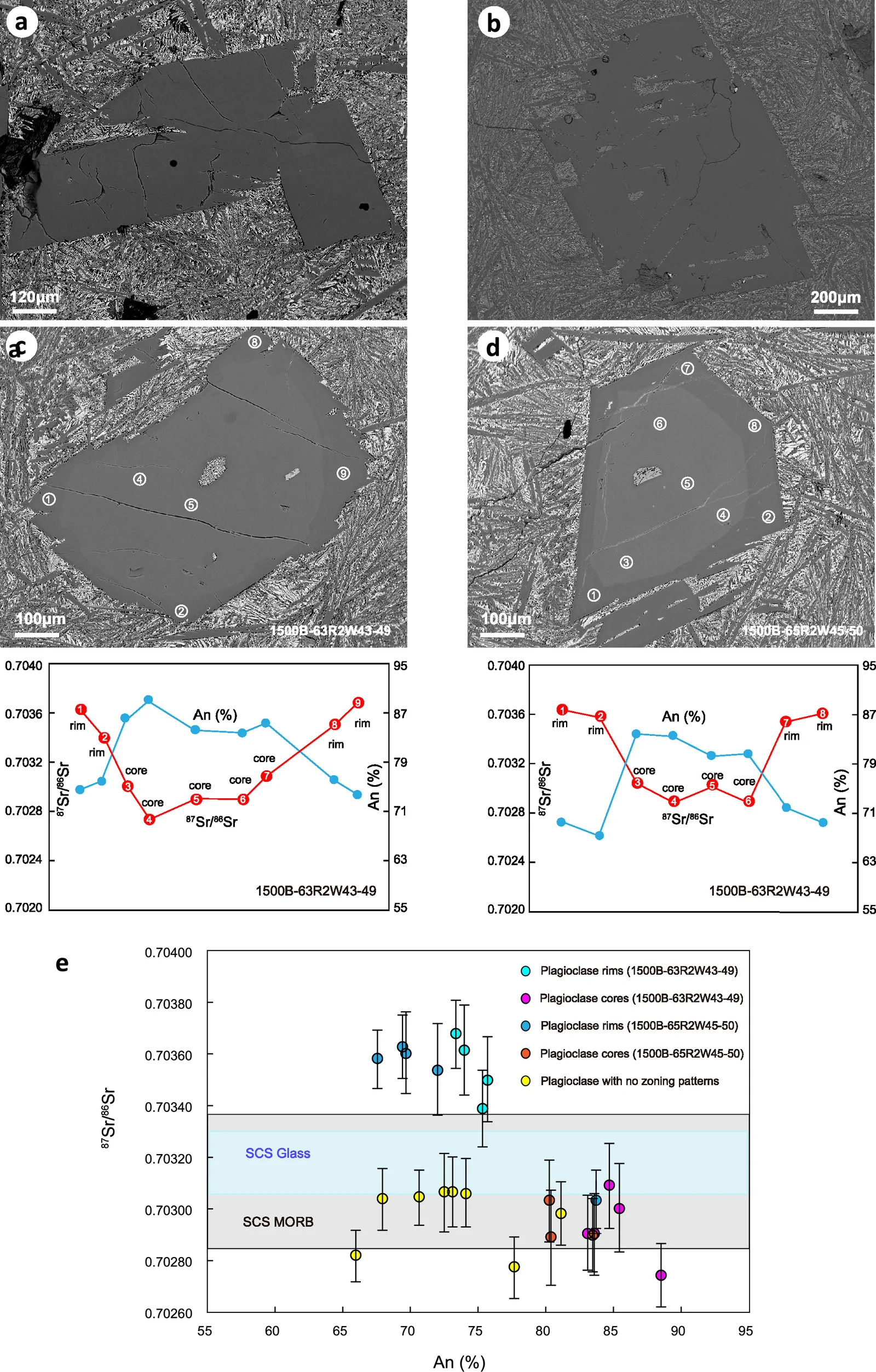
**Images and compositions of plagioclase phenocrysts and glasses from SCS MORB samples**. (a-b) Backscattered images of the analyzed plagioclase phenocrysts with no zoning. (c-d) Backscattered images of the analyzed plagioclase phenocrysts with zoning showing in-situ sampling locations (circles with numbers), and variation in ^87^Sr/^86^Sr (red spots) and An % rim to core profiles (blue spots) from basalts at Site U1500B. (e) Variation of ^87^Sr/^86^Sr isotopes and An contents for plagioclase with normal or no zoning compared to SCS glasses and MORB. The Sr isotope data of SCS spreading stage MORB is from the GEOROC (http://georoc.mpchmainz.gwdg.de/georoc).

Sites U1500 and 1502 plagioclase phenocrysts were selected for in-situ Sr isotopic analysis (Fig. 5; Table S2). The in-situ isotopic analyses reveal significant inter- and intracrystalline disequilibria within individual samples (Fig. 5; Table S2). As shown in Fig. 3, there is a clear progression from relatively low ^87^Sr/^86^Sr ratios (0.70275–0.70310) in the high-An cores (80.4–88.6) towards relatively high ^87^Sr/^86^Sr ratios (0.70340–0.70369) in the low-An rims (67.7–75.8) (Fig. 5). The ^87^Sr/^86^Sr isotopic ratios of plagioclase with no zoning patterns from Sites U1500 and 1502 basalts are largely similar to those of plagioclase cores but lower than those of plagioclase rims (Fig. 5).

### 4.3 Glass compositions

Glass samples from Site U1500 on the northern SCS margin are characterized by relatively low alkali content (Na₂O + K₂O = 2.82–3.52 wt%) and are predominantly tholeiitic basalts (Fig. 6a), consistent with previously published lavas from SCS basement drilling [36]. These glasses exhibit relatively high MgO (6.7–6.85 wt%; Table S3) and Mg# values [[56], [57]] (Table S3), indicating minimal fractionation of mafic minerals. The trace element patterns of the volcanic glasses are more enriched than typical N-MORB but slightly less so than typical E-MORB (Fig. 6b). The H_2_O contents of SCS MORB glasses range from 0.49 to 0.80 wt.% (0.73 wt.% ave.) (Fig. 6; Table S3), generally higher than the typical values of global MORB (generally < 0.2%) [[37], [38], [39], [40], [41]]. The H₂O/Ce ratios of these glasses vary from ∼344 to 587, significantly higher than global MORBs (mostly < 250) and typical OIBs (mostly < 150) [[37], [38], [39], [40]] (Fig. 6). Chlorine contents of SCS MORB glasses range from 41 to 237 ppm, and the Cl/K ratios range from 0.02 to 0.11, with most falling within the range of fresh MORB glasses (Cl/K < 0.1) [42] (Fig. 6). The ^87^Sr/^86^Sr isotopic ratios of these glasses are largely consistent with those of previously reported SCS MORBs (Fig. 5).

**Fig. 6:**
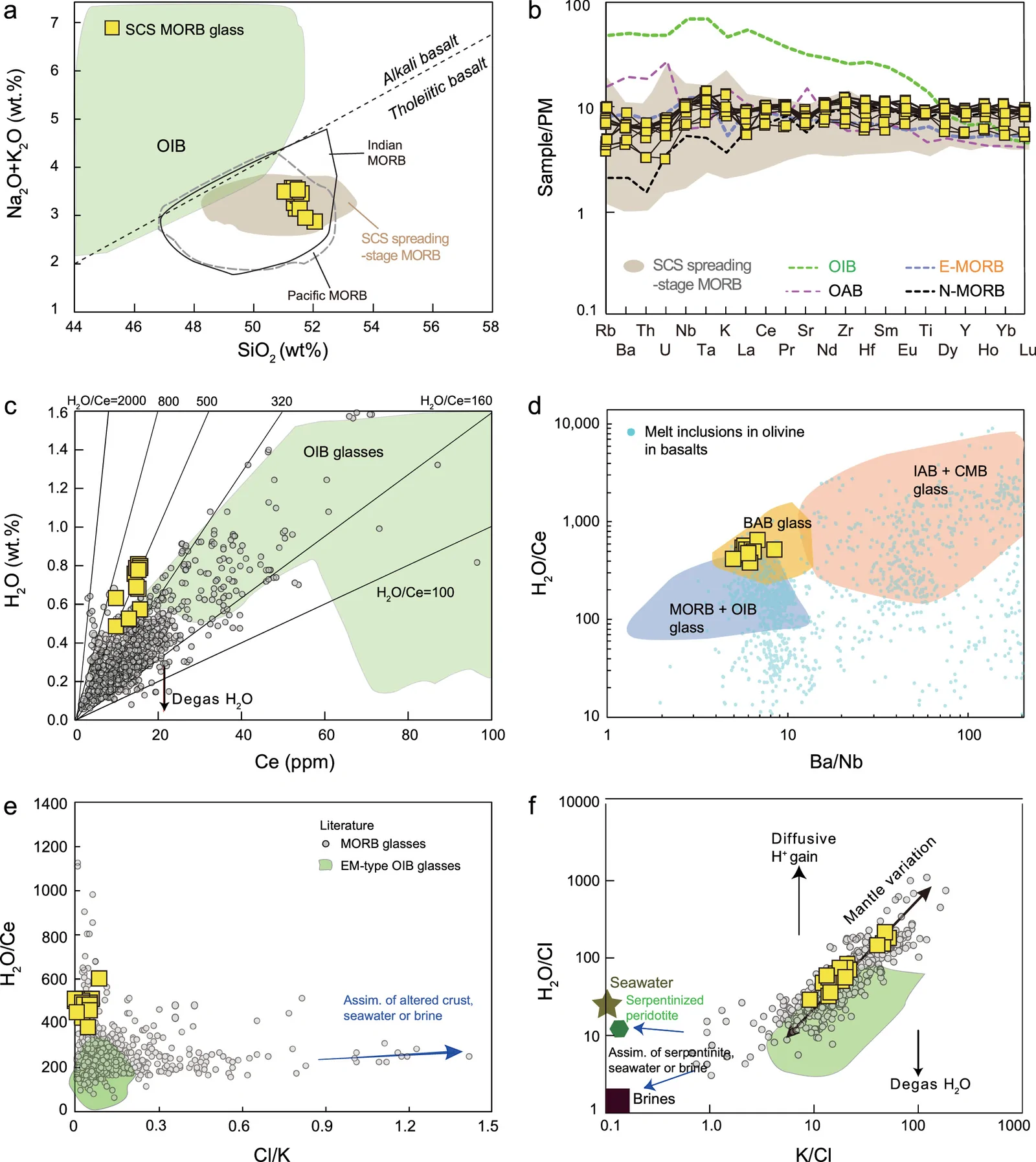
**Compositions of glasses from SCS MORB samples.** (a) Total alkalis (Na_2_O + K_2_O) versus SiO_2_ classification plot. (b) Spider diagram of SCS glass normalized to primitive mantle [71]. (c) H_2_O versus Ce. (d) H_2_O/Ce versus Ba/Nb. (e) H_2_O/Ce versus Cl/K. (f) H_2_O/Cl versus K/Cl. The data for glasses of mid-ocean ridge basalts (MORB), ocean island basalts (OIB), back-arc basin basalts (BAB), island-arc basalts (IAB), and continental margin basalts and andesites (CMB), melt inclusions in olivine in global basalts (small dots), N-MORB, E-MORB and OIB are from GEOROC database: http://georoc.mpch-mainz.gwdg.de/georoc/.

## 5 Discussion

### 5.1 Evidence for variable crustal assimilation

A notable feature of this study is the wide range of U-Pb ages observed in zircons from South China Sea (SCS) basalts at IODP Sites U1500 and U1502, spanning from 66 to 2740 Ma (Figs. 3, 4). These U-Pb ages significantly predate the eruption ages of their host lavas (∼30–34 Ma) [16], suggesting that the zircon grains are likely exotic or inherited xenocrysts. The zircons from SCS MORBs at Sites U1500 and U1502 are characterized by high concentrations of U, Th, and Y, as well as elevated U/Yb ratios, which contrast with the low concentrations of Th, U, and Y found in zircons from oceanic crust, mantle peridotites, and kimberlites [[43], [44], [45]], but resemble those of the continental crust [46,47] (Fig. 4). Furthermore, these zircons exhibit negative εHf(t) values ranging from −18 to −3.3 (Fig. 4), further supporting their continental crust origin. Together, these observations indicate that the zircons would be incorporated into the melt during the assimilation-fractional crystallization process and are more consistent with a continental crustal affinity beneath Sites 1500 and 1502.

Additionally, Sr isotopic disequilibrium between plagioclase crystals, and core-to-rim variations within individual crystals provide further evidence for assimilation during magma ascent. At Site U1500, plagioclase crystals in basalts exhibit core-to-rim variations in ^87^Sr/^86^Sr, with lower ratios in the crystal cores compared to the rims (Fig. 5). This clearly indicates that the SCS basalt magmas, irrespective of the mantle source characteristics, were modified by open-system processes occurring at crustal depths.

An origin involving the assimilation of magma with low ^87^Sr/^86^Sr by crust material with high ^87^Sr/^86^Sr is more plausible and consistent with the abrupt decrease in An from cores to rims (Fig. 5) [48]. This suggests that the magma from which the plagioclase rims grew was progressively more contaminated than that from which the cores originated. Altered oceanic crust or seawater-derived brines are potential contaminants for generating intra-crystal ^87^Sr/^86^Sr heterogeneity, as seawater typically has higher ^87^Sr/^86^Sr values compared to most MORBs [49].

We can assess the potential role of seawater assimilation through Cl and Cl/K systematics. Given that seawater is highly enriched in Cl and Cl/K compared to mantle-derived basalts, the addition of even small amounts of seawater, seawater-derived brines, or seawater-altered ocean crust would significantly elevate Cl and Cl/K [50]. However, the Cl/K ratios observed in the glass from our samples fall largely within the typical mantle range (below ∼0.1) [51] (Fig. 6). The modeling calculations (Fig. 7) also indicate that the assimilation of seawater-derived components is unlikely to explain observed variation in ^87^Sr/^86^Sr and trace element compositions in our samples. Instead, the continental crust, with its enriched ^87^Sr/^86^Sr signature [52], may be a more plausible contaminant, and its assimilation could account for the observed intra-crystal ^87^Sr/^86^Sr heterogeneity in the SCS plagioclases (Fig. 5).

**Fig. 7:**
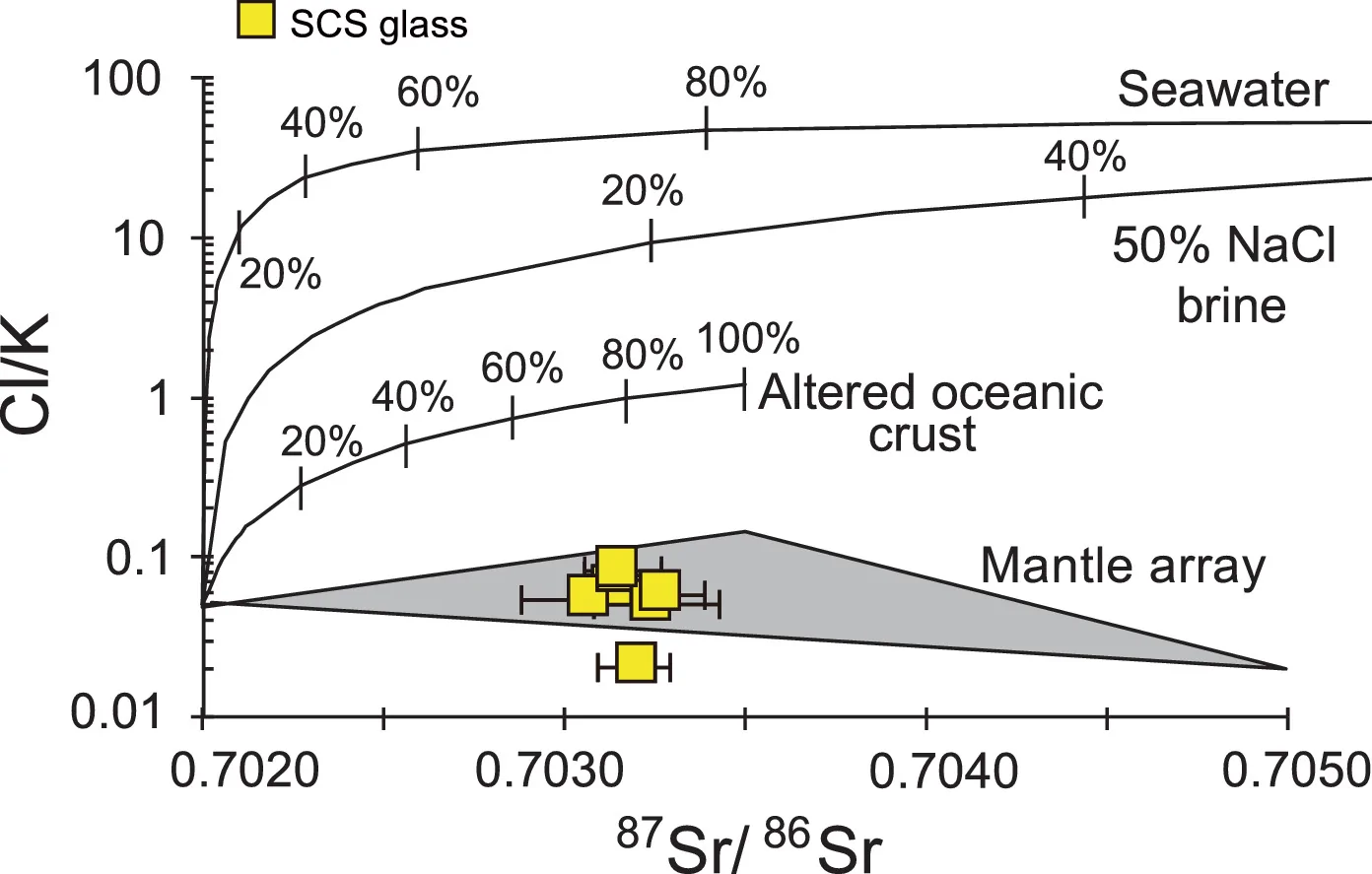
**Variation of ^87^Sr/^86^Sr isotopes and Cl/K for glasses. SWIR, Southwest Indian Ridge; JdF, Juan de Fuca Ridge; Blanco, BlancoTransform Zone**. ^87^Sr/^86^Sr values are averaged plagioclase, and Cl/K values are averaged plagioclase-hosted melt inclusions from the same samples, with bars showing total measured variation within plagioclase Sr isotopes and Cl/K [72].

As noted above, zircons with ages ranging from 66 to 2740 Ma were found in the basalts at IODP Sites U1500 and U1502 (Figs. 3, 4). The U-Pb ages and Hf isotope characteristics of the zircons in the SCS basalts (Fig. 4) broadly resemble those of detrital zircons from the Cathaysian Block of South China [53,54]. These findings suggest that the zircons were likely incorporated from the continental lithosphere beneath the SCS during magma ascent and/or within the magma chamber, implying the presence of continental crust beneath Sites U1500 and U1502.

### 5.2 Continental-ocean transitional zone structure beneath the northern SCS

The nature of the lithosphere beneath the northern SCS margin is crucial for understanding the formation mechanisms and processes of the SCS. Early interpretations of MCS profiles suggested that the COT of the northern SCS was magma-poor, potentially underlain by exhumed and serpentinized mantle [12]. Alternatively, seismic data and drilling results from IODP Expeditions 367/368/368X suggest the presence of a narrow COT separating the continental and oceanic domains in the northern SCS [9,10,55,56]. However, these findings cannot completely exclude the potential existence of exhumed mantle beneath the drilling areas of IODP Expeditions 367/368/368X. For example, while fresh MORBs have been recovered at Sites U1500 and U1502, a thin igneous carapace may be expected to overlie serpentinized exhumed mantle, similar to the west Iberia rifted margin [13], or igneous additions occur following exhumation and serpentinization of the mantle, as seen in the Vavilov and Marsili sub-basins of the Tyrrhenian Sea [57]. Our new results, together with published IODP drilling data of Larsen et al. [10], support the interpretation that the basement of the northern SCS COT is composed of extended continental crust, modified by igneous additions. Seismic and drilling data indicate that Ridge A is primarily underlain by the continental crust, with localized evidence of late Eocene to early Oligocene (30–34 Ma) MORB magmatism in its eastern part (Site U1502) [10]. This interpretation is further corroborated by our new geochemical data. The discovery of old zircon xenocrysts in basalts from Site 1502, derived from the South China continental lithosphere, implies the existence of continental crust beneath Ridge A of the northern SCS margin.

Crustal seismology and magnetic anomalies indicate the presence of a volcanic basement at least 2 km thick, with an early Oligocene age (magnetic Chron C11n, ∼30 Ma), as sampled at Site U1500 beneath Ridge B of the northern SCS margin [10,16]. However, it does not unequivocally mean that Ridge B is underlain by full oceanic crust, as a thick volcanic basement is adequate to produce the observed magnetic anomaly, even if it is largely underlain by remnants of continental lithosphere. Our new drilling data provide evidence for the presence of continental crust beneath Ridge B, based on the identification of plagioclases with isotopic signatures characteristic of continental lithosphere and old zircon xenocrysts in basalts at Site U1500. The Ridge B and C exhibit remarkable lateral continuity and smooth basement surfaces. Ridge B shares key features with Ridge C, in contrast to the continental Ridge A, forming a continuous extension into the younger, more mature oceanic igneous crust of the SCS. This suggests that beneath Ridge B, there is likely a transitional lithosphere, marking the shift from continental crust to mature oceanic crust. Our results thus support a rapid transition to the oceanic crust in a narrow region of the northern SCS passive margin [10], contradicting the magma-poor margin model that involves mantle exhumation [12].

### 5.3 Implications for marginal-sea rifting process

The North Atlantic lithosphere, exposed on the western and eastern sides of the Labrador Sea, was rifted apart between 60 and 55 Ma, accompanied by the eruption of flood basalts [58]. Although broad passive margins developed along both the northern and southern SCS continental margins, similar to those of the Atlantic Ocean, the SCS showed a different pattern in the transition from continental breakup to initial seafloor spreading [10]. Previous drilling results and seismic data indicate that the northern margin of the SCS experienced rapid lithosphere extension without mantle exhumation [10,59], facilitating decompression melting within the asthenosphere and the generation of MORB-type magmatism from a normal-temperature mantle. Wang et al. [60] suggested that this unique evolution of the SCS results from late Mesozoic to early Tertiary rifting episodes along an Andean-type proto-South China continental margin, contrasting with the intra-continental rifting followed by seafloor spreading observed in the Atlantic. An important question is what controls the different patterns of plate-edge rifting from intra-plate rifting.

Compared to Iberia-Newfoundland margins, the SCS is surrounded by several subduction systems, bounded by the Sumatra subduction zone in the west, Java subduction zone in the south, and Pacific Ocean-Philippine Sea subduction zones in the east [61]. The multiple subduction events surrounding the SCS may have carried abundant hydrous fluids/melts into the lithosphere prior to the SCS formation [62], making its mantle probably different from the north Atlantic rifted margins. The hydration of the lithospheric mantle is probably associated with the infiltration of water-rich fluids or the incorporation of hydrous melts during subduction. It is reasonable to speculate that the integrity of the lithosphere mantle had been significantly modified by regionally multiple subduction events surrounding the SCS since late Cretaceous-early Cenozoic [63] (Fig. 8). This is consistent with the volumes of Island arc-type volcanism developed in the stage of the SCS plate-edge rifting, in which partial melting of a wet and fertilized lithospheric mantle resulted [64]. Rehydration, through fluid or hydrous melt metasomatism, can significantly reduce the viscosity of the continental lithosphere, weakening it and making it more susceptible to removal by the convective asthenosphere [65,66]. In some cases, hydration-driven weakening (hydro-weakening) results in continental breakup and rifting [67]. Based on our new data, the SCS MORB glasses have H_2_O content (0.73 wt.% ave.), much higher than typical values of global MORB (H_2_O generally < 0.2%), and H_2_O/Ce ratios from ∼344 to 587, being considerably higher than global MORB glasses (mostly < 250) and typical OIB (mostly < 150) [[37], [38], [39], [40]] (Fig. 6; Table S3). This study argues that the hydration of the lithospheric mantle is also likely caused by wet upwelling of the MTZ components triggered by deep subduction of the Pacific/Paleo-SCS/ Indo-Australian oceanic lithosphere [25,68,69] (Fig. 8). This furthermore implies that the rifting of continental lithosphere is largely confined to its deepest parts, with fluid and melt ingress from below playing an important role in creating zones of persistent lithospheric weakness. This is also consistent with our previous inference that the lithosphere beneath the northern SCS underwent a large scale of extension and thinning but no mantle exhumation [9,10,59].

**Fig. 8:**
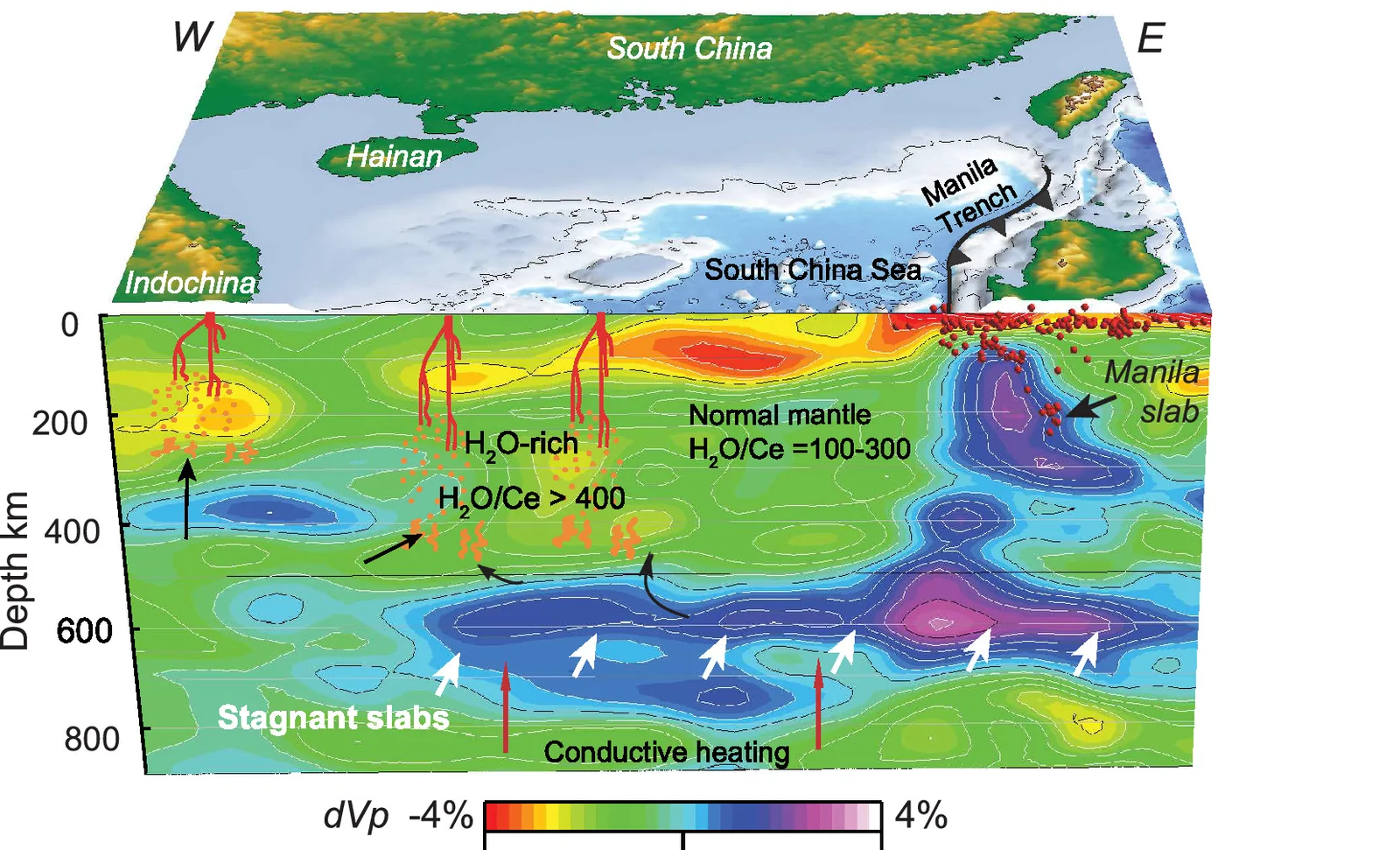
**Cartoon illustrating origin and recycling of water related to continental lithosphere thinning and rifting**. Partially molten upwelling plume from the mantle transition zone and captures materials enriched water. Water is supplied into the transition zone with the subducted oceanic lithosphere altered by seawater. Mantle structure beneath the South China Sea shown by FWEA18 full waveform tomography P-wave perturbations [73]. Tomographic fast anomalies indicate a swath of sub-horizontal subducted oceanic lithosphere beneath the SCS around 500–700 km depths, representing trapped subducted crust from previous subduction events.

## 6 Conclusion

This study provides new perspectives on the tectonic and magmatic processes driving the continental thinning and rifting of the SCS, with a particular focus on the COT zone. The notable disequilibria in Sr isotopes within plagioclase phenocrysts, coupled with the presence of zircons spanning from the Cretaceous to the Archean, provide compelling evidence for the presence of continental crust beneath the northern SCS margin at Sites 1500 and 1502. The co-existence of continental and oceanic crust within a narrow zone suggests a rapid transition from continental to oceanic lithosphere, challenging the notion of a simple extensional process and supporting a more complex tectonic scenario. The high-water content (average 0.73 wt.%) observed in SCS basalts, significantly exceeding that of typical mid-ocean ridge basalts, indicates prior hydration of the mantle, likely through the infiltration of hydrous fluids and melts from the mantle transition zone. This hydrous addition may have contributed to the weakening of the lithospheric root, facilitating the early-stage lithospheric thinning and rifting that ultimately led to the breakup of the SCS. These findings provide new insights into the lithospheric architecture of passive margins and the mechanisms driving continental breakup.

## Declaration of competing interest

The authors declare that they have no conflicts of interest in this work.

## Appendix Supplementary materials

Image, application 1
